# Cancer Stem Cells from Tumor Cell Lines Activate the DNA Damage Response Pathway after Ionizing Radiation More Efficiently Than Noncancer Stem Cells

**DOI:** 10.1155/2019/7038953

**Published:** 2019-04-03

**Authors:** Heriberto Abraham Valencia-González, Graciela Ruíz, Elizabeth Ortiz-Sánchez, Alejandro García-Carrancá

**Affiliations:** ^1^Programa de Maestría y Doctorado en Ciencias Bioquímicas, Facultad de Química, Universidad Nacional Autónoma de México (UNAM), Ciudad de México, Mexico; ^2^Laboratorio de Virus y Cáncer, Unidad de Investigación Biomédica en Cáncer, Instituto de Investigaciones Biomédicas, Universidad Nacional Autónoma de México & Instituto Nacional de Cancerología, Secretaría de Salud, Ciudad de México, Mexico

## Abstract

Recently, a subpopulation of tumor cells, called cancer stem cells (CSC), has been characterized, and these have emerged as a major topic in cancer research. CSC are proposed to repair DNA damage more efficiently than the rest of tumor cells, resisting chemotherapy or radiotherapy and causing clinical recurrence and metastasis. We aimed to determine the molecular basis of radioresistance and first compared the response to ionizing radiation (IR) between cancer stem cell-enriched cultures grown as spheres and conventional tumor cell line cultures grown as monolayer, from HeLa and MCF-7 cancer cell lines. To verify that our sphere cultures were enriched in CSC, we evaluated the double staining of CD49f and ALDH activity for HeLa cells by flow cytometry. We then evaluated whether differences could exist in sensor elements in the DNA damage response pathway among these cultures. We found that CSC cultures showed less sensitivity to radiation than conventional tumor cell line cultures. We observed a higher baseline expression of activated response sensor proteins of DNA damage, such as ATM, H2A.X, and PARP1, in untreated CSC cultures. These findings provide the first evidence, to our knowledge, that DNA damage response sensor proteins are present and preferentially activated in CSC, as opposed to the bulk of cells in monolayer cultures. Likewise, they provide the basis for biological differences in response to IR between CSC and other tumor cell populations. Understanding the DNA damage response pathway may provide therapeutic targets to sensitize CSC to cytotoxic therapies to improve current cancer treatments.

## 1. Introduction

Cancer is a disease of genetic and epigenetic alterations, which is highlighted as the central mechanisms of tumor progression in the multistep carcinogenesis model. Despite advances in surgical capabilities and chemotherapy strategies, a substantial proportion of patients continue to die from recurrent or chemotherapy-resistant disease. Such are the cases of cervical and breast cancers, where approximately 35% of women diagnosed with cervical cancer have recurrent disease, and 90% of these occur within 3 years of initial treatment [1], and women with breast cancer who are not diagnosed or treated early have a higher risk of dying from this disease [2].

Increasing evidence has suggested the existence of a subset of cells, called cancer stem cells (CSC) or cancer-initiating cells, which are distinct from the bulk of tumor cells and which are responsible for the long-term maintenance of tumor growth in several cancers. CSC can self-renew, drive tumorigenesis, are naturally resistant to chemotherapeutic agents, and might be responsible for tumor recurrence after therapy.

For studying and the obtaining of CSC cultures, the sphere-forming assay has been used, which serves as functional assay reported for enrichment in undifferentiated cells with enhanced tumor initiation ability from cancer cell lines [3, 4] and which has been used successfully to isolate and characterize CSC from several cancers such as brain [5], lung [6, 7], and breast [8–11].

To identify these CSC populations, CD49f and ALDH activity has been employed as markers. CD49f is an integrin *α*6 transmembrane protein that has been identified in germinal cells from embryonic stages to somatic cells in adult organisms [12]. CD49f works as laminin receptor and activates key signaling pathways for the invasion and migration of carcinoma cells [13, 14]. Also, high expression of CD49f has been associated with poor prognosis in cervical cancer patients [15]. On the other hand, ALDH is a polymorphic enzyme involved in the oxidation of aldehydes into carboxylic acids and may have a role in the early differentiation of stem cells, oxidizing retinol into retinoic acid [16]. ALDH was recognized as a marker of CSC, has been associated with chemoresistance in breast cancer [16, 17], and has been reported as enhancing the self-renewal and differentiation potentials in cervical cancer stem cells [16, 18, 19].

The mechanisms of CSC resistance to therapy include, among other aspects, an overexpression of DNA repair proteins [20–22]. The most harmful and most intensely studied lesions in DNA are double-strand breaks (DSB), which are caused by ionizing radiation (IR) during radiotherapy. The cellular response to DSB, known as the DNA damage response pathway, is developed through a series of steps involving sensor, transducer, and effector proteins [23, 24]. DSB are first detected by sensors as Ataxia Telangiectasia Mutated (ATM), which might recognize the DNA lesion itself or the chromatin alterations caused by DSB. Then, transducers such as H2A.X and PARP1 are recruited to the damage site, which serve to assemble the DSB repair complex at the site-of-damage and that activate downstream signaling to convey the DSB signal to the effectors. ATM is a kinase protein, a member of the phosphatidylinositol 3-kinase-related family of protein kinases. ATM transmits the message via various means, including phosphorylation of proteins such as the histone variant H2A.X, p53, and checkpoint kinase 2 [25]. PARP1 is a chromatin-associated protein that participates in various biological functions such as cell proliferation and apoptosis, as well as DNA repair. In damaged DNA, PARP1 is activated and catalyzes the polyADPribosylation of diverse nuclear proteins, including elements of DNA repair pathways [26]. Some reports show an adverse prognosis in breast cancer regarding the overexpression of PARP1 [27, 28].

CSC are proposed to repair DNA damage more efficiently than other tumor cells, resisting the therapies and causing clinical recurrence and metastasis after therapy. Our aim was to determine the differences that exist in elements in the DNA damage response after exposure to IR between cancer stem cell-enriched (CSC) cultures growing as spheres and conventional tumor cell lines growing as monolayer. Our findings provide the first evidence, to our knowledge, that response sensor proteins of DNA damage are present and activated preferentially in CSC cultures, as opposed to the bulk of cells in conventional cultures, and that they provide the basis for the biological differences in the response to IR between CSC and other tumor cell populations.

## 2. Materials and Methods

### 2.1. Cell Cultures

The human cervical cancer cell line HeLa (authenticated by STR DNA profiling at University of Colorado DNA Sequencing and Analysis Core according to the report number DP0297 issued by the university) was maintained and grown as monolayer (HeLa ML) using Dulbecco's modified Eagle's medium (DMEM) (Gibco®), and the human breast cancer cell line MCF-7 (ATCC) was maintained and grown as monolayer (MCF-7 ML) using DMEM F-12 (Gibco®), with both culture media containing 10% fetal bovine serum (FBS) (Gibco®), the antibiotics penicillin and streptomycin (Gibco®), and incubated at 37°C in a humidified atmosphere containing 5% CO_2_. The sphere-forming assay of cell lines was performed using MammoCult™ medium (Stem Cell Technologies) with serum replacement, hydrocortisone, heparin, and antibiotics according to supplier's instructions (Stem Cell Technologies). The sphere cultures were obtained with 3,000 cells/mL of HeLa (HeLa SP) or MCF-7 (MCF-7 SP) cell lines, which was seeded onto a 100 mm ultra-low attachment culture dish (Corning) using 7 mL of MammoCult™ medium. Then, this was incubated for 4 days at 37°C in a humidified atmosphere containing 5% CO_2_. SP cultures were used on day 4 of growth for the assays, and ML cultures were used at 80% of confluence. The SP cultures are the cancer stem cell-enriched cultures and the ML cultures are the conventional tumor cell line cultures.

### 2.2. Protein Extraction and Western Blot

Total protein of cultures was obtained with lysis buffer (50 mM Tris Baze, 5 mM EDTA, 133 mM NaCl, 1 mM PMSF, and 1% Triton X-100). Quantification was performed using the Pierce™ BCA Protein Assay kit (Thermo Scientific). The already extracted proteins were preserved at -70°C. 50 *μ*g of protein was separated using sodium dodecyl sulfate-polyacrylamide gel electrophoresis (SDS-PAGE) and transferred onto nitrocellulose membranes. Then, these were blocked with 5% bovine serum albumin (BSA) in 0.05% phosphate-buffered saline solution-tween 20 (PBST) for 1 h at room temperature, followed by incubation with primary antibody against ATM (Abcam, ab78), pATM (Abcam, ab36810), PARP1 (Santa Cruz Biotechnology, sc-8007), GAPDH (Santa Cruz Biotechnology, sc-48167), and *β*-actin (Santa Cruz Biotechnology, sc-47778) at 4°C overnight. Subsequently, the membranes were incubated with secondary antibody goat anti-mouse IgG-HRP (Santa Cruz Biotechnology, sc-2005) or donkey anti-goat IgG-HRP (Santa Cruz Biotechnology, sc-2020) diluted in 5% BSA for 1 h at room temperature and washed with PBST. Immunodetection was performed using Immobilon™ Western chemiluminescent HRP substrate (Millipore, WBKLS0500) and visualized with C-DiGit equipment (Li-Cor). Data were presented as relative expression protein levels normalized for *β*-actin or GAPDH protein.

### 2.3. Ionizing Radiation

ML and SP cell cultures were harvested and resuspended in DMEM and in Mammocult medium, respectively. Cultures were irradiated at doses of 0–10 Gy using a self-contained Gammacell 1000 Elite Nordion Cesium 137 Irradiator with an emission rate of 4.056 Gy/min (Instituto de Investigaciones Biomédicas, UNAM). The cultures were maintained on ice prior to irradiation; immediately after irradiation, cells were returned to the incubator for recovery until the appropriate time point. To perform the assay, the cultures were exposed to a dose of IR that affected the survival fraction at around 0.5, for HeLa ML was 1.5 Gy and for HeLa SP was 6.0 Gy.

### 2.4. Flow Cytometry

Cancer stem cell markers: HeLa ML and HeLa SP cultures were suspended in flow buffer (0.05% BSA, 2 mM EDTA in PBS). For CD49f protein, the cell suspension was stained with anti-CD49f-PE (BD Biosciences, 555736) for 30 min, then washed and maintained with cold flow buffer until reading. For the ALDH assay, the Aldefluor™ kit (Stem Cell Technologies, 01700) was performed according to supplier's instructions. For the double staining with CD49f and ALDH activity, the first staining was performed with the ALDH assay followed by staining with anti-CD49f-PE which maintained these cold and protected from light until reading. The reading of the samples was done with the Beckman Coulter Cytometer (UIMEO-IMSS, México); the CD49f-positive cells were visualized by the phycoerythrin channel (FL-2), and the ALDH activity-positive cells were visualized by the fluorescein isothiocyanate channel (FL-1). The data were analyzed with FlowJo® software.

### 2.5. Cell Cycle Analysis

After IR treatment, cell cultures were incubated for 0-24 h, fixed with 2% paraformaldehyde for 5 min, washed with PBS, and permeabilized with methanol at -20°C. The cells were suspended in staining buffer (5 *μ*g/mL propidium iodide (Sigma, P4170), 10 *μ*g/mL RNAse A (Invitrogen, 12091-02), 0.5% Triton X-100 (USB, 22686), and PBS) for 40 min, read on an Attune AV Flow Cytometer (BD, LabNalCit IIB-UNAM), and analyzed with FlowJo® software.

### 2.6. Clonogenic Assay

After treatment with doses of 0-10 Gy IR, 500 single cells/well of ML cultures were seeded on 6-well microplates (Corning), three wells per condition; on day 14, the colonies formed were fixed with 4% paraformaldehyde and stained with 0.5% crystal violet. For SP cultures, 100 single cells/well were seeded on 96-well ultra-low attachment microplates (Corning), 12 wells per condition; on day 7, only the spheres formed were counted. Colonies or spheres larger than 70 *μ*m were qualified as deriving from a single cloning cell. Plating efficiency (PE) and survival fraction (SF) after the dose were calculated using equations (1) and (2). The clonogenic assay for ML cultures was read on day 14 to render the growth of colonies more evident; the SP cultures were read at day 7 because, after this time, indistinguishable agglomerates are formed. The median lethal dose (LD50) was obtained employing an exponential mathematical model.

(1) $$\begin{array}{c} \text{PE}=\frac{\text{Number of colonies or spheres by control}}{\text{Number of seeded cells}}, \end{array}$$

(2) $$\begin{array}{c} \text{SF}=\frac{\text{Number of colonies or spheres formed after treatment}}{\left(\text{Number of seeded cells}\right)\left(\text{PE}\right)}. \end{array}$$

### 2.7. Immunofluorescence

After the IR treatment, the cell cultures were incubated at 0.5 and for 1 h in their respective media at 37°C, with 5% CO_2_. After incubation, the cultures were harvested, fixed with 2% paraformaldehyde for 5 min, and suspended in PBS. Individual cells were attached to the slide by centrifugation with CytoSpin Rotofix 32A (Hettich). The cells on the slide were immunostained with the *γ*H2A.X (Abcam, ab81299) and pATM (Abcam, ab36810) antibodies. In brief, cells were blocked with blocking solution (5% SFB, 0.6% Triton X-100, PBS) for 1 h, washed with PBS, incubated with the antibodies overnight at 4°C, washed with PBS, incubated with secondary fluorescent antibody goat anti-rabbit IgG-FITC (Santa Cruz Biotechnology, sc-2012) or goat anti-mouse IgG-Fluorescein (Santa Cruz Biotechnology, sc-2010) for 2 h, washed with PBS, and mounted using Vectashield mounting medium with DAPI (Vector Laboratories, H-1200). The images were acquired with an Olympus IX71 inverted microscope or a Nikon A1R+STORM confocal microscope (Unidad de Microscopía at the Instituto de Investigaciones Biomédicas, UNAM). The images were analyzed with ImageJ and NIS-Elements Viewer software.

### 2.8. Statistical Analysis

Statistical analysis was performed using a two-tailed Student *t*-test and one-way analysis of variance (ANOVA) with post hoc Tukey test by GraphPad Prism statistical software (ver. 5.0). *P* values < 0.01 were considered statistically significant.

## 3. Results

### 3.1. Obtaining Cancer Stem Cell-Enriched Cultures as Spheres

**Figure 1:**
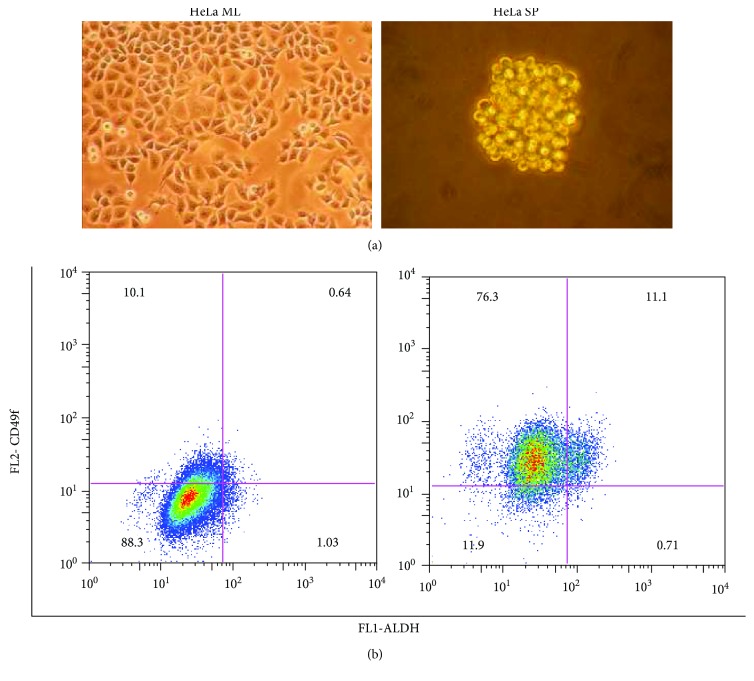
HeLa SP are positive for cancer stem cell markers. (a) Microscopy of cultures derived from the HeLa cell line growing as monolayer (HeLa ML) and as spheres (HeLa SP), objectives 20x and 40x, respectively. (b) Flow cytometry analysis of HeLa ML and HeLa SP cultures for CD49f and high ALDH activity, with the gates representing the percentage of cell populations positive for staining. For HeLa SP, 87% of cells were positive for CD49f, nearly 12% were positive for high ALDH activity, and for both markers, 11% were positive. In HeLa ML, 88% of cells were negative for both markers, while less than 1% were positive for the latter.

With the sphere-forming assay, we obtained the sphere cultures grown under the nonadherent conditions that we have previously used this method to obtain cancer stem cell-enriched (CSC) cultures from tumor cell lines grown as monolayer, which were considered conventional tumor cell cultures. Employing this strategy, we obtained CSC cultures of the HeLa cell line grown as spheres (HeLa SP). We found that 87% of the cells of HeLa SP cultures were CD49f-positive, while 80% of the cells of HeLa grown as monolayer (HeLa ML) were CD49f-negative (Figure 1). The expression of both CD49f and ALDH activity was present in 11% of the population grown as spheres, while only 0.64% of the population grown as monolayer were positive. Also, we performed a sphere-forming assay to obtain CSC cultures from the MCF-7 breast cancer cell line. In this case, the growth by formation of spheres is sufficient to obtain CSC cultures.

### 3.2. Cancer Stem Cells Display Less Sensitivity to Ionizing Radiation

**Figure 2:**
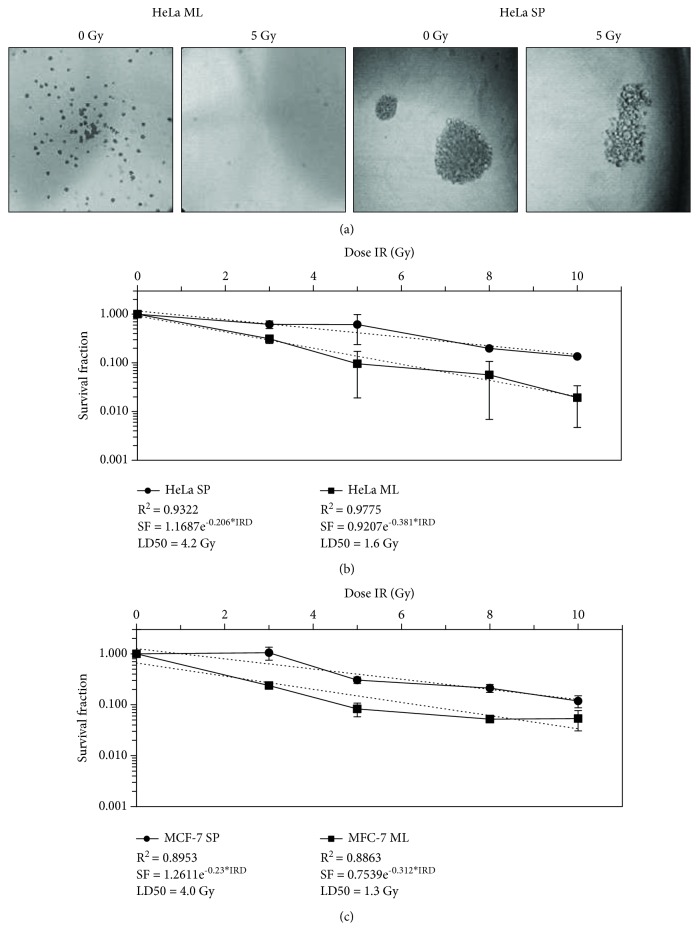
Sphere cultures from HeLa and MCF-7 cells are less sensitive to ionizing radiation. (a) Clonogenic assay indicated that up to a 5 Gy IR dose, HeLa ML died while HeLa SP continued to proliferate. Objective 4x and 40x, respectively. (b) Survival data after ionizing radiation doses showed that HeLa SP and (c) MCF7 SP have a higher survival fraction (SF) than HeLa ML and MCF-7 ML cultures. The median lethal dose (LD50) was calculated using an exponential mathematical model; for HeLa ML, this was 1.6 Gy, for HeLa SP, this was 4.2 Gy, for MCF-7 ML, this was 1.3 Gy, and for MCF-7 SP, this was 4.0 Gy. Data shown are represented as mean ± standard error of the mean (SEM) of two independent experiments. IRD: ionizing radiation dose.

The determination of the sensitivity of ionizing radiation (IR) demonstrated that sphere cultures were less sensitive to radiation than the monolayer cultures of HeLa and MCF-7 cell lines. As expected, both growth conditions of the cell lines revealed a progressive decrease in survival with an increasing dose of IR, but CSC cultures exhibited less sensitivity to IR. ML cultures did not survive after having been exposed to doses greater than 5 Gy, while SP cultures survived up to the 6 Gy dose (Figure 2(a)). Through an exponential mathematical model, it was possible to obtain the median lethal dose (LD50) of each culture; for HeLa ML this was 1.6 Gy, for HeLa SP this was 4.2 Gy (Figure 2(b)), for MCF-7 ML this was 1.3 Gy, and for MCF-7 SP, this was 4.0 Gy (Figure 2(c)). The LD50 of the CSC cultures was higher than the LD50 of conventional tumor cell line cultures. The plating efficiency (PE) of HeLa ML was 47.2% and 39.0% for HeLa SP, while the PE of MCF-ML was 90% and 10% for MCF-7 SP.

We report the clonogenic assay at days 7 and 14 of growth after radiation for SP and ML cultures, respectively. However, the number of colonies formed on day 7 by ML cultures did not change on day 14. Growth up to day 14 evidenced the size of the colonies.

### 3.3. Progression of the Cell Cycle Showed No Differences among Cultures after Ionizing Radiation

We examined possible changes in the cell cycle of HeLa SP and ML cultures up to 24 h after radiation. Both ML and SP cultures stopped the cell cycle in the G2/M phase 12 h after exposure to the IR dose; we observed an accumulation of 64% of the cell population in HeLa ML and of 83% in HeLa SP (Figure S1). It is very important to highlight that cancer stem cell-enriched cultures, after having stopped their cell cycle, continued to proliferate. Notwithstanding that ML cultures restored the cell cycle, they could not continue to proliferate later in time, in agreement with the clonogenic assay.

### 3.4. Activation of ATM and PARP1 Is More Efficient in Cancer Stem Cells after Ionizing Radiation

**Figure 3:**
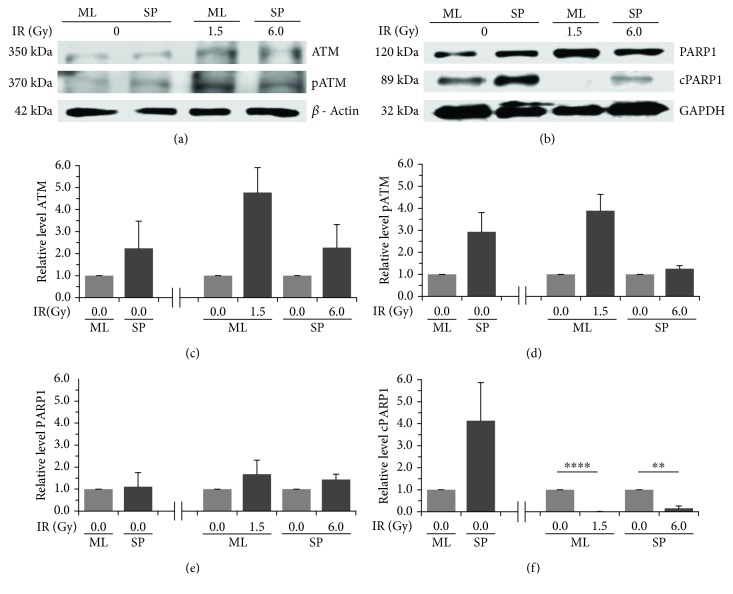
ATM and PARP1 are present and activated in cancer stem cells before and after ionizing radiation. (a) Representative immunoblot showing overall expression of ATM and phospho ATM (pATM) in SP and ML cultures before and 3 h after exposure to ionizing radiation (IR). (b) Representative immunoblot showing overall expression of PARP1 and cleaved PARP1 (cPARP1) in HeLa SP and HeLa ML cultures before and 3 h after exposure to IR. (c) HeLa SP cultures had a higher level of ATM protein than HeLa ML cultures without exposure to IR. After exposure to IR, ATM expression increased in both cultures. (d) HeLa SP cultures had a higher level of pATM than HeLa ML cultures without exposure to IR. After exposure to IR, only HeLa ML cultures had an increase of pATM. (e) HeLa SP and HeLa ML cultures exhibited no differences in the level of PARP1 protein before and after exposure to IR. (f) HeLa SP cultures had a higher level of cPARP1 than HeLa ML before exposure to IR. After exposure to IR, both cultures had a decreased level of cPARP1; however, HeLa SP continued to express cPARP1. *β*-Actin and GAPDH were used as loading control. The relative level of these proteins to *β*-Actin and GAPDH was analyzed by densitometry. The normalized level of proteins was expressed as mean ± standard error of the mean (SEM) at least two independent experiments.

We evaluated the expression of ATM and PARP1 in HeLa SP cultures without IR and compared these with HeLa ML cultures without IR. We found that HeLa SP cultures had a higher level than HeLa ML cultures of ATM and of phospho ATM (pATM), the latter the activated form of ATM. When HeLa ML cultures were exposed to a 1.5 Gy IR dose, the ATM and pATM proteins increased between 2- and 3-fold (Figures 3(a), 3(c), and 3(d)). On the other hand, HeLa SP and HeLa ML cultures without an IR dose did not exhibit differences in the level of the PARP1 protein, while HeLa SP and HeLa ML demonstrated the same level. However, the level of cleaved PARP1 protein (cPARP1) was 3-fold higher in HeLa SP. When HeLa ML and HeLa SP were exposed to the previously mentioned IR dose, the level of PARP1 protein continued for 3 h without change between treated and untreated cultures; in the opposite manner, the level of cPARP1 protein was different between treated and untreated cultures. HeLa SP continued to have more cPARP1 protein than HeLa ML after exposure to their IR dose (Figures 3(b), 3(e), and 3(f)). It is important to highlight that sphere cultures at a dose of 6 Gy of IR respond in a similar way to that of monolayer cultures at a 1.5 Gy IR dose.

### 3.5. Cancer Stem Cells Possess an Active DNA Damage Response Pathway

**Figure 4:**
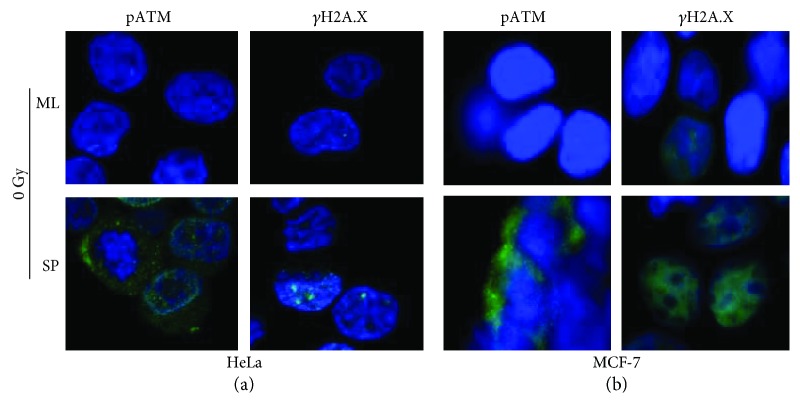
Cancer stem cells have baseline expression of pATM and *γ*H2A.X. Immunofluorescence staining for pATM and *γ*H2A.X protein (green). Visualization of nuclei by DAPI (blue). (a) HeLa SP cultures expressed pATM and *γ*H2A.X in baseline levels. (b) MCF-7 SP cultures expressed pATM and *γ*H2A.X in baseline levels. The representative images of the microscopy analysis indicate that pATM and *γ*H2A.X proteins are expressed in baseline levels in CSC cultures. Objective 60x.

**Figure 5:**
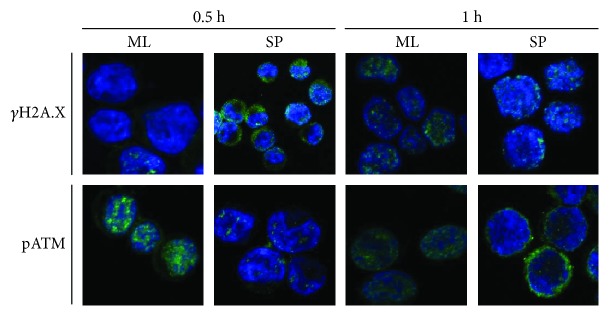
HeLa SP overexpress pATM and *γ*H2A.X after ionizing radiation. Localization of pATM and *γ*H2A.X expression after 0.5 h and 1 h exposure to ionizing radiation in HeLa SP and HeLa ML using anti-pATM antibody (green) and anti-*γ*H2A.X antibody (green). Visualization of nuclei by DAPI (blue). The representative images of the microscopy analysis indicated that *γ*H2A.X is activated always in SP cultures, while in ML cultures, the activation of *γ*H2A.X began 1 h after exposure to 1.5 Gy IR dose. Objective 60x.

The presence of phosphorylated H2A.X (*γ*H2A.X) and phosphorylated ATM (pATM) protein in HeLa ML and HeLa SP was detected by immunofluorescence. Interestingly, HeLa SP has a baseline expression of these proteins compared with HeLa ML without exposure to radiation (Figure 4(a)). Also, the presence of *γ*H2A.X increased with IR treatment in both types of cultures. With a 1.5 Gy dose of IR, both cultures increased the presence of *γ*H2A.X and pATM 1 h after radiation. Following the kinetic of expression for up to 1 h, both *γ*H2A.X and pATM were higher after irradiation in HeLa SP and HeLa ML cultures. However, prior to IR treatment, the level of pATM protein did not appear in monolayer cultures, while in sphere cultures, this was expressed in the baseline condition and increased with radiation (Figure 5). The signal of *γ*H2A.X was strong in HeLa SP after radiation, but this was lower at 0.5 h after radiation in HeLa ML, while increasing 1 h later. pATM was identified in HeLa SP and HeLa ML cultures under the same conditions as *γ*H2A.X. A time-course immunofluorescence for pATM demonstrated a strong induction after IR at 0.5 h and 1 h. As expected, pATM and *γ*H2A.X were identified mainly in MCF-7 SP cultures without exposure to IR, as in HeLa cells (Figure 4(b)).

## 4. Discussion

Cancer stem cells, responsible for resistance to therapy and tumor recurrence in several types of cancer, are being intensively studied to identify the molecular and cellular bases of events that block the complete elimination of tumors. According to different reports, a strategy used to obtain cancer stem cell-enriched (CSC) cultures comprises the sphere-forming assay. In order to characterize these populations, cervical cancer stem cell markers, such as CD49f and ALDH activity, have been reported [29–35]. Thus, in our work, we performed this method to obtain CSC cultures from a cervical cancer cell line and we performed, to our knowledge for the first time, double staining for CD49f protein and high ALDH enzyme activity in order to characterize these. Likewise, with the sphere-forming assay, we obtained CSC cultures from the MCF-7 breast cancer cell line [3]. With our results, we confirmed that our sphere culture model is composed of cells with a specific phenotype and enzymatic activity different from that of conventional tumor cell cultures (CD49 and high ALDH activity).

Recently, it has been reported that CSC cultures have an overexpression of genes involved in their DNA repair machinery for the double-strand break (DSB) and to exhibit radioresistance [34]. Therefore, we evaluated the sensitivity to ionizing radiation (IR) by inducing DNA DSB in CSC cultures. As expected, we observed that the LD50 of CSC cultures is higher than the LD50 of conventional tumor cell cultures. This result is congruent with those of other previous works, such as in one in which glioblastoma cells with stemness characteristics exhibited radioresistance [36]. According to our results, sphere cultures are less sensitive to IR than conventional tumor cell cultures from HeLa and MCF-7 cell lines.

To identify the stage of DNA damage sensing in the DNA damage response (DDR) pathway, SP and ML cultures were exposed at an IR dose near to that of their LD50, which affected cell survival by around 50%. Interestingly, we found that key proteins of DDR, such as pATM and *γ*H2A.X, had a higher expression prior to IR treatment compared with monolayer. This is curious because the phosphorylation of ATM and H2A.X entertains the consequence of an alteration in the continuity of the DNA strand [37–39]. The expression of *γ*H2A.X under baseline conditions in HeLa SP could be explained by means of previous works reporting that numerous H2A.X molecules in the chromatin surrounding each DSB are phosphorylated within minutes after irradiation; these are known as “*γ*H2A.X foci.” These *γ*H2A.X foci are thought to serve as a platform for the recruitment of DNA repair and checkpoint signaling factors [40, 41].

In addition to this, we provide additional evidence concerning the beginning of the DDR pathway in CSC from cervical cancer, where ATM, H2A.X, and PARP1 are key elements involved in the detection of DNA damage. We found that CSC cultures overexpress these proteins after IR compared with conventional tumor cell line cultures. Interestingly, we show here, to our knowledge for the first time, that CSC cultures exhibit an elevated expression of DNA damage sensors, which are active basally, and this could explain the high capacity of CSC for DNA repair. This new evidence confirms the idea that CSC possess an efficient DNA damage response ready to detect DNA damage quickly and reveals a more efficient response and a lower sensitivity to IR of cancer stem cells.

HeLa SP and HeLa ML exhibited high levels of pATM and *γ*H2A.X 1 h after irradiation. However, when we performed the evaluation on cells without radiation, we observed the baseline expression of pATM and *γ*H2A.X only in HeLa SP and their absence in HeLa ML cultures. This indicates a more efficient response and a lower sensitivity to the IR of the cancer stem cells than conventional tumor cell line cultures. These findings suggest that radioresistance is specifically associated with the stemness state [34, 36, 42–44] suggesting that cancer stem cell-enriched cultures are more efficient in detecting DNA damage due to IR. Cell cycle regulation is an important response after DNA damage, and previously, this evaluation had been carried out 24 h after radiation, but no differences were observed between monolayer and sphere cultures. Both cultures restore the cell cycle, but only CSC remained clonogenic, even after a higher IR dose. This confirms that pATM is a sensor of damage in CSC and that it plays a central role in the cellular response to IR, activating cell cycle checkpoints that lead to DNA damage-induced arrest in DNA repair.

We have observed that these cultures have a greater tumorigenic potential both *in vitro* and *in vivo*, also demonstrating greater resistance to DSB caused by a drug [45]. Cell death does not necessarily mean an advantage regarding the therapeutic result in radiation therapy against cancer [46]. This could be important in terms of understanding the possible implications in tumor recurrence and in managing the treatment. Despite the frequent use of chemotherapy, at present, cellular resistance to antineoplastic treatment is one of the main problems in the effective control of some types of tumors. Unfortunately, there continue to be tumors for which chemotherapy has not achieved great success: tumors with a good response to treatment commonly become resistant to drugs, irremediably causing the death of patients. Therefore, with our results, we provide more evidence to support the role of CSC in radioresistance to cervical cancer.

## 5. Conclusions

Our study provides, to our knowledge, the first evidence that cancer stem cells from cancer-derived cell lines exhibit radioresistance due to the increased presence of components from the DNA damage response, specifically sensor proteins. We additionally found new evidence that CSC cultures had increased the expression of ATM, pATM, PARP1, and *γ*H2A.X after radiation treatment. These results support critical roles for cancer stem cells in determining tumor response to therapy. The study of the DNA damage response pathways may provide therapeutic targets to sensitize cancer stem cells to radiation therapies and to prevent tumor recurrence. Our findings may be of great value for the development of therapies using as targets DNA damage response proteins.

## Data Availability

The data (all the results) used to support the findings of this study are included within the article.

## Abbreviations

CSC: Cancer stem cells
ML: Monolayer
SP: Spheres
DSB: Double-strand break
IR: Ionizing radiation
DNA: Deoxyribonucleic acid
LD50: Median lethal dose
DDR: DNA damage response.

## Supplementary Materials

Supplementary Materials Cell cycle analysis of HeLa SP and ML cultures at different time points after radiation. Both ML and SP cultures were either not incubated or incubated for 3, 6, 9, 12, and 24 hours after two different IR doses, fixed with 2% paraformaldehyde for 5 min, washed with PBS, and permeabilized with methanol at -20°C for 10 minutes. Cells were suspended in staining buffer (5 *μ*g/mL propidium iodide (Sigma, P4170), 10 *μ*g/mL RNAse A (Invitrogen, 12091-02), 0.5% Triton X-100 (USB, 22686), and PBS) for 40 min, fluorescence read on an Attune AV Flow Cytometer (BD, LabNalCit IIB-UNAM), and analyzed with FlowJo® software. Figure S1: no differences exist between the percentage of SP and ML cells at different cell cycles.

## References

[1] Pectasides D., Kamposioras K., Papaxoinis G., Pectasides E. (2008). Chemotherapy for recurrent cervical cancer. *Cancer Treatment Reviews*.

[2] Justo N., Wilking N., Jonsson B., Luciani S., Cazap E. (2013). A review of breast cancer care and outcomes in Latin America. *The Oncologist*.

[3] Grimshaw M. J., Cooper L., Papazisis K. (2008). Mammosphere culture of metastatic breast cancer cells enriches for tumorigenic breast cancer cells. *Breast Cancer Research*.

[4] Ponti D., Costa A., Zaffaroni N. (2005). Isolation and *in vitro* propagation of tumorigenic breast cancer cells with stem/progenitor cell properties. *Cancer Research*.

[5] Lathia J. D., Gallagher J., Heddleston J. M. (2010). Integrin alpha 6 regulates glioblastoma stem cells. *Cell*.

[6] Corominas-Faja B., Oliveras-Ferraros C., Cuyàs E. (2013). Stem cell-like ALDH^bright^ cellular states in EGFR-mutant non-small cell lung cancer: a novel mechanism of acquired resistance to erlotinib targetable with the natural polyphenol silibinin. *Cell Cycle*.

[7] Lundholm L., Hååg P., Zong D. (2013). Resistance to DNA-damaging treatment in non-small cell lung cancer tumor-initiating cells involves reduced DNA-PK/ATM activation and diminished cell cycle arrest. *Cell Death & Disease*.

[8] Cariati M., Naderi A., Brown J. P. (2008). Alpha-6 integrin is necessary for the tumourigenicity of a stem cell-like subpopulation within the MCF7 breast cancer cell line. *International Journal of Cancer*.

[9] Dontu G., Abdallah W. M., Foley J. M. (2003). In vitro propagation and transcriptional profiling of human mammary stem/progenitor cells. *Genes and Development*.

[10] Goel H. L., Gritsko T., Pursell B. (2014). Regulated splicing of the Α6 integrin cytoplasmic domain determines the fate of breast cancer stem cells. *Cell Reports*.

[11] Schott A. F., Landis M. D., Dontu G. (2013). Preclinical and clinical studies of gamma secretase inhibitors with docetaxel on human breast tumors. *Clinical Cancer Research*.

[12] Krebsbach P. H., Villa-Diaz L. G. (2017). The role of integrin Α6 (CD49f) in stem cells: more than a conserved biomarker. *Stem Cells and Development*.

[13] Belkin A. M., Stepp M. A. (2000). Integrins as receptors for laminins. *Microscopy Research and Technique*.

[14] Yoon S. O., Shin S., Lipscomb E. A. (2006). A novel mechanism for integrin-mediated Ras activation in breast carcinoma cells: the Α_6_
*β*
_4_ integrin regulates ErbB2 translation and transactivates epidermal growth factor receptor/ErbB2 signaling. *Cancer Research*.

[15] Hou T., Zhang W., Tong C. (2015). Putative stem cell markers in cervical squamous cell carcinoma are correlated with poor clinical outcome. *BMC Cancer*.

[16] Ginestier C., Hur M. H., Charafe-Jauffret E. (2007). ALDH1 is a marker of normal and malignant human mammary stem cells and a predictor of poor clinical outcome. *Cell Stem Cell*.

[17] Tanei T., Morimoto K., Shimazu K. (2009). Association of breast cancer stem cells identified by aldehyde dehydrogenase 1 expression with resistance to sequential paclitaxel and epirubicin-based chemotherapy for breast cancers. *Clinical Cancer Research*.

[18] Fukamachi H., Seol H. S., Shimada S. (2013). CD49f^high^ cells retain sphere-forming and tumor-initiating activities in human gastric tumors. *PLoS One*.

[19] Xie Q., Liang J., Rao Q. (2016). Aldehyde dehydrogenase 1 expression predicts chemoresistance and poor clinical outcomes in patients with locally advanced cervical cancer treated with neoadjuvant chemotherapy prior to radical hysterectomy. *Annals of Surgical Oncology*.

[20] Dalerba P., Cho R. W., Clarke M. F. (2007). Cancer stem cells : models and concepts. *Annual Review of Medicine*.

[21] Ishii H., Iwatsuki M., Ieta K. (2008). Cancer stem cells and chemoradiation resistance. *Cancer Science*.

[22] Maugeri-Saccà M., Vigneri P., De Maria R. (2011). Cancer stem cells and chemosensitivity. *Clinical Cancer Research*.

[23] Blanpain C., Mohrin M., Sotiropoulou P. A., Passegue E. (2011). DNA-damage response in tissue-specific and cancer stem cells. *Cell Stem Cell*.

[24] Maugeri-Sacca M., Bartucci M., De Maria R. (2012). DNA damage repair pathways in cancer stem cells. *Molecular Cancer Therapeutics*.

[25] Sancar A., Lindsey-Boltz L. A., Ünsal-Kaçmaz K., Linn S. (2004). Molecular mechanisms of mammalian DNA repair and the DNA damage checkpoints. *Annual Review of Biochemistry*.

[26] Gilabert M., Launay S., Ginestier C. (2014). Poly(ADP-ribose) polymerase 1 (PARP1) overexpression in human breast cancer stem cells and resistance to olaparib. *PLoS One*.

[27] Gonçalves A., Finetti P., Sabatier R. (2011). Poly(ADP-ribose) Polymerase-1 MRNA expression in human breast cancer: a meta-analysis. *Breast Cancer Research and Treatment*.

[28] von Minckwitz G., Müller B. M., Loibl S. (2011). Cytoplasmic poly(adenosine diphosphate-ribose) polymerase expression is predictive and prognostic in patients with breast cancer treated with neoadjuvant chemotherapy. *Journal of Clinical Oncology*.

[29] Bortolomai I., Canevari S., Facetti I. (2010). Tumor initiating cells: development and critical characterization of a model derived from the A431 carcinoma cell line forming spheres in suspension. *Cell Cycle*.

[30] Feng D., Peng C., Li C. (2009). Identification and characterization of cancer stem-like cells from primary carcinoma of the cervix uteri. *Oncology Reports*.

[31] Gu W., Yeo E., McMillan N., Yu C. (2011). Silencing oncogene expression in cervical cancer stem-like cells inhibits their cell growth and self-renewal ability. *Cancer Gene Therapy*.

[32] Li X.-s., Xu Q., Fu X.-y., Luo W.-s. (2014). ALDH1A1 overexpression is associated with the progression and prognosis in gastric cancer. *BMC Cancer*.

[33] Liu S.-Y., Zheng P.-S. (2013). High aldehyde dehydrogenase activity identifies cancer stem cells in human cervical cancer. *Oncotarget*.

[34] López J., Poitevin A., Mendoza-Martínez V., Pérez-Plasencia C., García-Carrancá A. (2012). Cancer-initiating cells derived from established cervical cell lines exhibit stem-cell markers and increased radioresistance. *BMC Cancer*.

[35] Ortiz-Sánchez E., Santiago-López L., Cruz-Domínguez V. B. (2016). Characterization of cervical cancer stem cell-like cells: phenotyping, stemness, and human papilloma virus co-receptor expression. *Oncotarget*.

[36] Bao S., Wu Q., McLendon R. E. (2006). Glioma stem cells promote radioresistance by preferential activation of the DNA damage response. *Nature*.

[37] Bakkenist C. J., Kastan M. B. (2003). DNA damage activates ATM through intermolecular autophosphorylation and dimer dissociation. *Nature*.

[38] Lavin M. F., Kozlov S. (2007). ATM activation and DNA damage response. *Cell Cycle*.

[39] Shiloh Y., Ziv Y. (2013). The ATM protein kinase: regulating the cellular response to genotoxic stress, and more. *Nature Reviews Molecular Cell Biology*.

[40] Bonner W. M., Redon C. E., Dickey J. S. (2008). *Γ*H2AX and cancer. *Nature Reviews Cancer*.

[41] Fernandez-Capetillo O., Lee A., Nussenzweig M., Nussenzweig A. (2004). H2AX: the histone guardian of the genome. *DNA Repair*.

[42] Baumann M., Krause M., Hill R. (2008). Exploring the role of cancer stem cells in radioresistance. *Nature Reviews Cancer*.

[43] Kumazawa S., Kajiyama H., Umezu T. (2014). Possible association between stem-like hallmark and radioresistance in human cervical carcinoma cells. *The Journal of Obstetrics and Gynaecology Research*.

[44] Shen L., Huang X., Xie X., Su J., Yuan J., Chen X. (2014). High expression of SOX2 and OCT4 indicates radiation resistance and an independent negative prognosis in cervical squamous cell carcinoma. *Journal of Histochemistry & Cytochemistry*.

[45] Ruíz G., Valencia-González H. A., León-Galicia I., García-Villa E., García-Carrancá A., Gariglio P. (2018). Inhibition of RAD51 by SiRNA and resveratrol sensitizes cancer stem cells derived from HeLa cell cultures to apoptosis. *Stem Cells International*.

[46] Mirzayans R., Andrais B., Scott A., Wang Y., Murray D. (2013). Ionizing radiation-induced responses in human cells with differing TP53 status. *International Journal of Molecular Sciences*.

