# Supplementary material for: Cancer Stem Cells from Tumor Cell Lines Activate the DNA Damage Response Pathway after Ionizing Radiation More Efficiently Than Noncancer Stem Cells
Source: Stem Cells Int. 2019 Apr 3;2019:7038953. doi: 10.1155/2019/7038953 (PMC6470433; doi:10.1155/2019/7038953)
Supplement: Supplementary Materials — Cell cycle analysis of HeLa SP and ML cultures at different time points after radiation. Both ML and SP cultures were either not incubated or incubated for 3, 6, 9, 12, and 24 hours after two different IR doses, fixed with 2% paraformaldehyde for 5 min, washed with PBS, and permeabilized with methanol at -20°C for 10 minutes. Cells were suspended in staining buffer (5 μg/mL propidium iodide (Sigma, P4170), 10 μg/mL RNAse A (Invitrogen, 12091-02), 0.5% Triton X-100 (USB, 22686), and PBS) for 40 min, fluorescence read on an Attune AV Flow Cytometer (BD, LabNalCit IIB-UNAM), and analyzed with FlowJo® software. Figure S1: no differences exist between the percentage of SP and ML cells at different cell cycles. [file 7038953.f1.docx]

**Figure S1**

**Figure S1. No differences between SP and ML in cell-cycle.** SP and ML cultures stopped their progression in the cell-cycle at between 9 and 12 h after exposure to IR. However, both cultures recovered the cell-cycle after 12 h.
